# Human Astrocytes: Secretome Profiles of Cytokines and Chemokines

**DOI:** 10.1371/journal.pone.0092325

**Published:** 2014-04-01

**Authors:** Sung S. Choi, Hong J. Lee, Inja Lim, Jun-ichi Satoh, Seung U. Kim

**Affiliations:** 1 Medical Research Institute, Chung-Ang University College of Medicine, Seoul, Korea; 2 Department of Physiology, Chung-Ang University College of Medicine, Seoul, Korea; 3 Department of Bioinformatics and Molecular Neuropathology, Meiji Pharmaceutical University, Kiyose, Japan; 4 Division of Neurology, Department of Medicine, UBC Hospital, University of British Columbia, Vancouver, Canada; University of South Florida, United States of America

## Abstract

Astrocytes play a key role in maintenance of neuronal functions in the central nervous system by producing various cytokines, chemokines, and growth factors, which act as a molecular coordinator of neuron-glia communication. At the site of neuroinflammation, astrocyte-derived cytokines and chemokines play both neuroprotective and neurotoxic roles in brain lesions of human neurological diseases. At present, the comprehensive profile of human astrocyte-derived cytokines and chemokines during inflammation remains to be fully characterized. We investigated the cytokine secretome profile of highly purified human astrocytes by using a protein microarray. Non-stimulated human astrocytes in culture expressed eight cytokines, including G-CSF, GM-CSF, GROα (CXCL1), IL-6, IL-8 (CXCL8), MCP-1 (CCL2), MIF and Serpin E1. Following stimulation with IL-1β and TNF-α, activated astrocytes newly produced IL-1β, IL-1ra, TNF-α, IP-10 (CXCL10), MIP-1α (CCL3) and RANTES (CCL5), in addition to the induction of sICAM-1 and complement component 5. Database search indicated that most of cytokines and chemokines produced by non-stimulated and activated astrocytes are direct targets of the transcription factor NF-kB. These results indicated that cultured human astrocytes express a distinct set of NF-kB-target cytokines and chemokines in resting and activated conditions, suggesting that the NF-kB signaling pathway differentially regulates gene expression of cytokines and chemokines in human astrocytes under physiological and inflammatory conditions.

## Introduction

Astrocytes belong to one of three major types of neuroglia in the central nervous system (CNS) and play active roles in many neuronal functions: maintaining ion and pH homeostasis, promoting the synthesis and removal of neurotransmitters, providing glucose supply and antioxidant defense, and regulating synaptic activity by producing various cytokines, chemokines, growth factors, and metabolites, all of which act as a molecular coordinator of neuron-glia communication [1]. At the site of neuroinflammation, astrocyte-derived cytokines and chemokines play both neurotoxic (inflammatory) and neuroprotective (immunoregulatory) roles in the brains of human neurological diseases, such as multiple sclerosis (MS), Alzheimer's disease (AD), Parkinson's disease (PD), and HIV-1 associated dementia (HAD) [2]. However, at present, the exact profile of human astrocyte-derived cytokines and chemokines during inflammation remain mostly unclear, possibly attributable to the limited availability of non-malignant human astrocyte cell lines that serve as an *in vitro* model of normal human astrocytes. In the present study, we characterized the comprehensive profiles named “secretome” [3] of cytokines and chemokines derived from cultured normal human astrocytes, compared under resting and activated conditions by using a protein microarray.

## Materials and Methods

### Ethics statement

Fetal brain tissue was obtained from a 15 weeks human fetus and dissociated cells prepared as described previously [4], [5]. Human tissue collected for research purpose was approved by the Chung-Ang University Ethics Committee on Human Subject (Certificate #09-0041). Pregnant women gave written informed consents for clinical procedure and research use of the embryonic tissue in accordance with the declaration of Helsinki.

### Human astrocytes in culture

The brain tissue isolated from a fetus of 15 weeks gestation, was dissociated into single cells by incubation with 0.25% trypsin in phosphate-buffered saline (PBS) for 30 min, as described previously [4], [5]. Dissociated cells were suspended in the culture medium, composed of the Dulbecco's modified Eagle medium (DMEM) with high glucose (Invitrogen, Carlsbad, CA) supplemented with 10% fetal bovine serum (FBS) (Invitrogen) and 20 µg/mL gentamicin (Sigma, St Louis, MO). Dissociated cells were plated at 5×10^7^ cells/T75 flask and were grown in an incubator with 5% CO_2_ atmosphere. In primary brain cell cultures, all microglia freely floating in the medium were removed. After replating the cultures for three to five times by treatment with trypsin, most of neurons and oligodendrocytes underwent cell death and detached off, while astrocytes were firmly attached onto the flask surface, resulting in enrichment of highly purified astrocytes.

### Immunocytochemistry

Human astrocytes cultured on poly-L-lysine-coated Aclar plastic coverslips (9 mm in diameter) were fixed in methanol for 10 min at −20°C. The cells were incubated with primary antibodies specific for GFAP (1∶1,000, rabbit; Millipore, Billerica, MA), a cell type specific marker for astrocytes, or tubulin βIII antibody (1∶200, mouse, Millipore), a. neuron specific marker, for 48 hrs at 4°C followed by Alexa Fluor594-conjugated anti-rabbit IgG or anti-mouse IgG for 1 hr at room temperature (RT). For immunostaining for cell type markers for oligodendrocytes and microglia, astrocytes on coverslips were fixed in 4% paraformaldehyde for 2 min, washed in PBS, incubated in anti-galactocerebroside antibody (1∶4, mouse; Kim Lab), a oligodendrocyte cell type-specific marker, or anti-human CD68 antibody (1∶200, mouse, Millipore), a microglial marker, for 48 hrs at 4°C, followed by Alexa Fluor 594-conjugated anti-mouse IgG for 1 hr at RT. Cultures processed for immunocytochemistry were examined under an Olympus laser confocal fluorescence microscope.

### Cytokine and chemokine profiling

Human astrocytes were incubated in culture medium with or without inclusion of a mixture of 10 ng/mL recombinant human IL-1β (Peprotech, Rocky Hill, NJ) and 10 ng/mL recombinant human TNF-α (Peprotech). At 24 hours after treatment, the conditioned media were harvested and processed for profiling of cytokines and chemokines on the human cytokine array panel A (R&D system, Minneapolis, MN), which is capable of detecting a panel of 36 cytokines, chemokines, and soluble mediators, such as complement 5/5a, CD40 ligand, G-CSF, GM-CSF, GROα, I-309, sICAM-1, IFN-γ, IL-1α, IL-1β, IL-1ra, IL-2, IL-4, IL-5, IL-6, IL-8, IL-10, IL-12p70, IL-13, IL-16, IL-17, IL-17E, IL-23, IL-27, IL-32α, IP-10, I-TAC, MCP-1, MIF, MIP-1α, MIP-1β, Serpin E1, RANTES, SDF-1, TNFα, and sTREM-1 (Table 1). The array membranes were reacted with the mixture of conditioned media and the antibody cocktail for 18 hrs at 4°C. After several washing, they were briefly incubated with secondary antibodies conjugated with horseradish peroxidase (HRP). Then, the membranes were exposed to HRP substrate. The intensity of the reaction was quantified on the Da vinci imaging system (Seoul, Korea).

**Table 1:** Proteome profiler array of human cytokines/chmokines used in the present study.

Location	Control	Expression ratio in stimulated astrocytes
A3, A4	Complement Component 5/5a (C5/C5a)	Up
A5, A6	CD40 Ligand (CD154)	-
A7, A8	G-CSF (CSFβ, CSF-3)	Up
A9, A10	GM-CSF (CSFα, CSF-2)	Up
A11, A12	GROα (CXCL1)	Up
A13, A14	I-309 (CCL1)	-
A15, A16	sICAM-1 (CD54)	Up
A17, A18	IFN-γ (Type II IFN)	-
B3, B4	IL-1α (IL-1F1)	-
B5, B6	IL-1β (IL-1F2)	Up
B7, B8	IL-1ra (IL-1F3)	Up
B9, B10	IL-2	-
B11, B12	IL-4	-
B13, B14	IL-5	-
B15, B16	IL-6	Up
B17, B18	IL-8 (CXCL8)	Up
C3, C4	IL-10	-
C5, C6	IL-12 p70	-
C7, C8	IL-13	-
C9, C10	IL-16 (LCF)	-
C11, C12	IL-17	-
C13, C14	IL-17E	-
C15, C16	IL-23	-
C17, C18	IL-27	-
D3, D4	IL-32	-
D5, D6	IP-10 (CXCL10)	Up
D7, D8	I-TAC (CXCL11)	-
D9, D10	MCP-1 (CCL2)	Down
D11, D12	MIF (GIF, DER6)	Down
D13, D14	MIP-1α (CCL3)	Up
D15, D16	MIP-1β (CCL4)	-
D17, D18	Serpin E1 (PAI-1)	Up
E3, E4	RANTES (CCL5)	Up
E5, E6	SDF-1 (CXCL12)	-
E7, E8	TNF-α (TNFSF1A)	Up
E9, E10	sTREM-1	-

### Molecular network analysis

We imported Entrez Gene IDs corresponding to cytokine and chemokine genes into the Core Analysis tool of Ingenuity Pathways Analysis (IPA) (Ingenuity Systems; www.ingenuity.com). IPA is a commercial knowledgebase that contains information on approximately 3,000,000 biological and chemical interactions with definite scientific evidence. By uploading the list of Gene IDs, the network-generation algorithm identifies focused genes integrated in global molecular networks. IPA calculates the score p-value that reflects the statistical significance of association between the genes and the networks by Fisher's exact test. We considered p-value<0.05 as a significant association. The information on known NF-kB target genes was collected from web accessible databases constructed by Dr. Thomas Gilmore, Boston University (www.bu.edu/nf-kb/gene-resources/target-genes) and by Bonsai Bioinformatics, Laboratoire d'Informatique Fondamentale de Lille (LIFL), Université Lille 1 (bioinfo.lifl.fr/NF-KB), as described previously [6].

## Results

### Cytokine and chemokine profiles of human astrocytes in culture

**Figure 1:**
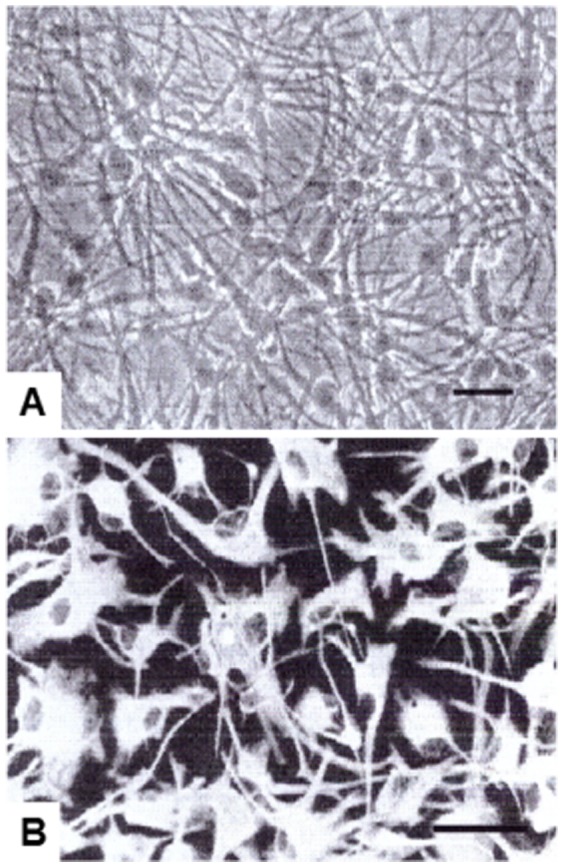
The purity of human astrocytes in culture exceeded 99% glial fibrillary acidic protein (GFAP) immunoreactivity-positive. Astocytes shown are at the normal non-stimulated resting state. A: Phase contrast microscopy. B: Immunostaning with anti-GFAP antibody.

**Figure 2:**
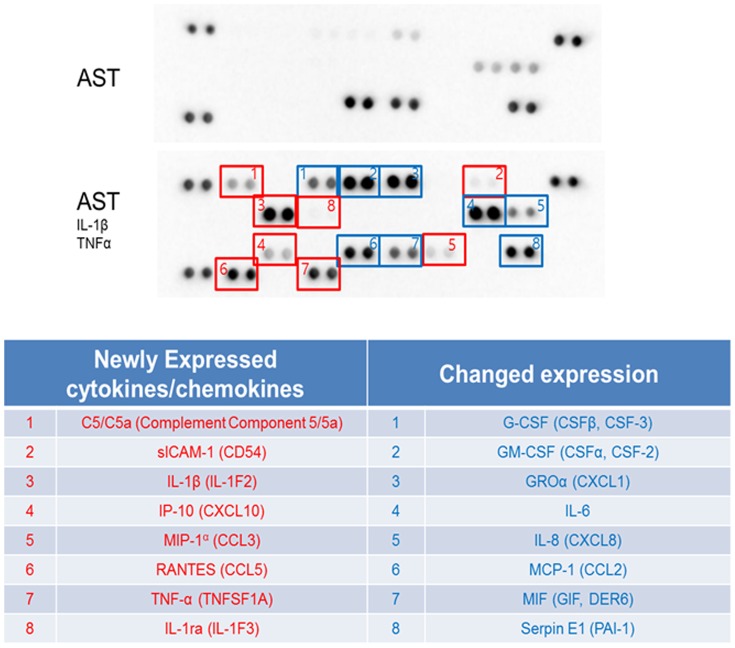
Proteome profiler array of human cytokines/chmokines. The array is capable of detecting a panel of 36 cytokines, chemokines, and soluble mediators. Top panel shows expression of cytokines/chemokines in resting unstimulated human astrocyrtes, and middle panel shows expression of cytkines/chemokines in human astrocytes stimulated with IL-1β and TNF-α. Items circled in red are newly expressed cytokines in activated astrocytes and ones circled in blue are cytokines changed expression in activated aqstrocytes. In bottom panel, newly expressed cytokines and cytokines changed expression are listed separately.

**Figure 3:**
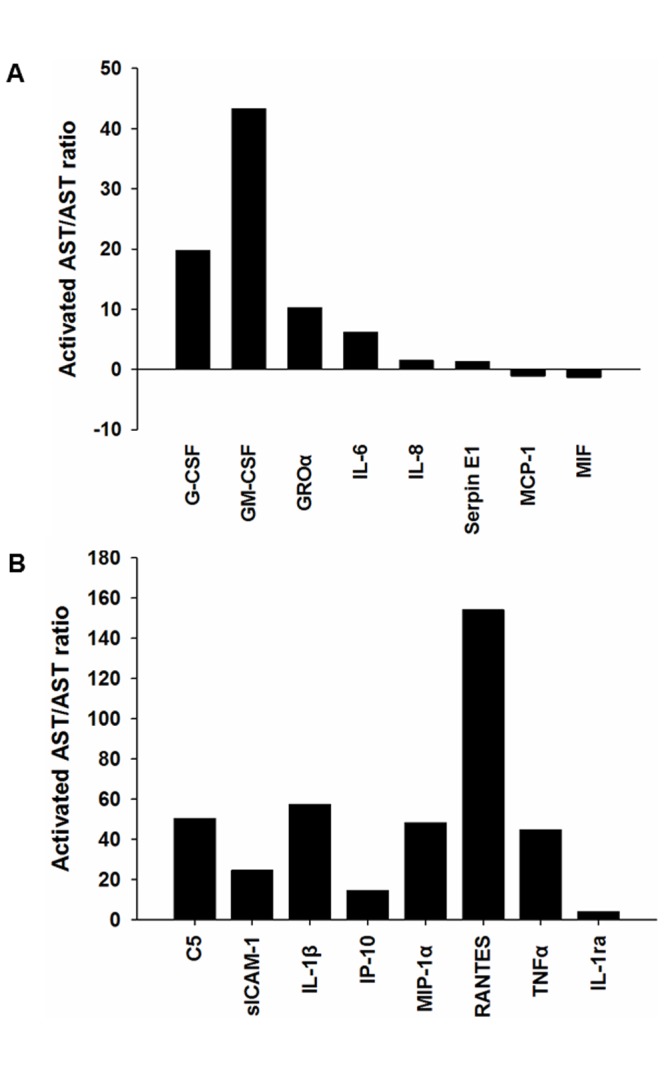
Cytokines/chemokines changed their secretion levels in human astrocytes stimulated with IL-1β and TNF-α. A: Forty five-fold increase in secertion of GM-CSF and twenty-fold increase in secretion of G-CSF are shown here. A minor reduction in expression of MCP-1 and MIF is also shown. B. Among the elevated levels of cytokines/chemokines in human astrocytes stimulated with IL-1β and TNF-α, upregulated expression of RANTES was the most prominent with more than 150-fold increase.

The purity of human astrocytes in culture exceeded 99% by GFAP labeling without any contamination of the cells expressing CD68 (microglia), galactocerebroside (oligodendrocytes) or tubulin βIII (neurons) (Fig. 1). Non-stimulated resting astrocytes incubated in the culture medium without addition of cytokines expressed 8 out of 36 cytokines and chemokines tested, such as G-CSF, GM-CSF, IL-6, GROα (CXCL1), IL-8 (CXCL8), MCP-1 (CCL2), MIF and serpin E1 (Table 2, Fig. 2). Other cytokines and chemokines were undetectable in the conditioned media of non-stimulated astrocytes (Table 3). Following a 24 hr-exposure to a mixture of IL-1β and TNF-α, the expression levels of 6 cytokines, including G-CSF, GM-CSF, IL-6, GROα, IL-8 and Serpin E1, were elevated substantially (Table 4, Figs. 2 and 3), whereas the levels of both MCP-1 and MIF showed a minor reduction (Table 5, Figs. 2 and 3). In astrocytes activated with IL-1β/TNF-α for 24 hrs, there was new indcution of cytokines and chemokines including IL-1β, IL-1ra, TNF-α, IP-10 (CXCL10), MIP-1α (CCL3), RANTES (CCL5), sICAM-1 (CD54) and complement component 5 (C5a) (Table 6, Figs. 2 and 3), suggesting an existence of the positive autoregulatory feedback loop for expression of IL-1β and TNF-α. Among them, upregulated expression of RANTES was the most prominent (Fig. 3B). By database search on known NF-kB target genes, nearly all cytokines and chemokines produced by non-stimulated and activated astrocytes are direct targets of the transcription factor NF-kB, except for C5a, IL-1ra, and MIF, although the genes encoding C5a and MIF have NF-kB binding sites in the promoter regions by literature search on PubMed [7], [8].

**Table 2:** Cytokines expressed in normal resting human astrocytes.

Gene	Genbank	Gene Name
G-CSF	NM_000759	colony stimulating factor 3 (granulocyte)
GM-CSF	NM_000758	colony stimulating factor 2
MCP-1 (CCL2)	NM_002982	chemokine (C-C motif) ligand 2
GROα (CXCL1)	NM_001511	chemokine (C-X-C motif) ligand 1
MIF	NM_002415	macrophage migration inhibitory factor
IL-6	NM_000600	interleukin 6
IL-8 (CXCL8)	NM_000584	interleukin 8
Serpin E1	NM_000602	plasminogen activator inhibitor type 1

**Table 3:** Cytokines absent in normal resting human astrocytes.

Gene	Genebank	Gene name
CD40 Ligand	NM_000074	CD40 ligand
I-309 (CCL1)	NM_002981	chemokine (C-C motif) ligand 1
IFN-γ (Type II IFN)	NM_000619	interferon, gamma
IL-1α (IL-1F1)	NM_000575	interleukin 1, alpha
IL-2	NM_000586	interleukin 2
IL-4	NM_000589	interleukin 4
IL-5	NM_000879	interleukin 5 (colony-stimulating factor, eosinophil)
IL-10	NM_000572	interleukin 10
IL-12 p70	NM_000882/	interleukin 12A (natural killer cell stimulatory factor 1)
IL-13	NM_002188	interleukin 13
IL-16	NM_172217	interleukin 16
IL-17	NM_002190	interleukin 17A
IL-17E	NM_022789	interleukin 25
IL-23	NM_016584	interleukin 23, alpha subunit p19
IL-27	NM_145659	interleukin 27
IL-32	NM_001012633	interleukin 32
I-TAC (CXCL11)	NM_005409	chemokine (C-X-C motif) ligand 11
MIP-1β (CCL4)	NM_002984	chemokine (C-C motif) ligand 4
SDF-1 (CXCL12)	NM_199168	chemokine (C-X-C motif) ligand 12
sTREM-1	NM_018643	triggering receptor expressed on myeloid cells 1

**Table 4:** Cytokines upregulated in human astrocytes following IL-1β/TNFα treatment.

Gene	Fold	Genbank	Gene Name
G-CSF	19.74	NM_000759	colony stimulating factor 3 (granulocyte)
GM-CSF	43.25	NM_000758	colony stimulating factor 2 (granulocyte-macrophage)
GROα (CXCL1)	10.27	NM_001511	chemokine (C-X-C motif) ligand 1
IL-6	6.09	NM_000600	interleukin 6
IL-8 (CXCL8)	1.47	NM_000584	interleukin 8
Serpin E1(PAI-1)	1.30	NM_000602	plasminogen activator inhibitor type 1

**Table 5:** Cytokines downregulated in human astrocytes following IL-1β/TNFα treatment.

Gene	Fold	Genbank	Gene Name
MCP-1 (CCL2)	−1.04	NM_002982	chemokine (C-C motif) ligand 2
MIF	−1.28	NM_002415	macrophage migration inhibitory factor

**Table 6:** Cytokines newly induced in human astrocytes following IL-1β/TNFα treatment.

Gene	Fold	Genbank	Gene Name
IL-1β	57.17	NM_000576	interleukin 1, beta
IL-1rα	3.72	NM_002182	interleukin 1 receptor accessory protein
TNFα	44.75	NM_000594	tumor necrosis factor (TNF superfamily, member 2)
sICAM-1	24.38	NM_000201	intercellular adhesion molecule 1 (CD54)
C5	50.25	NM_001735	complement component 5
IP-10 (CXCL10)	14.27	NM_001565	chemokine (C-X-C motif) ligand 10
MIP-1α (CCL3)	48.05	NM_002983	chemokine (C-C motif) ligand 3
RANTES (CCL5)	154.13	NM_002985	chemokine (C-C motif) ligand 5

### Molecular network of cytokine and chemokine secretome of human astrocytes

**Figure 4:**
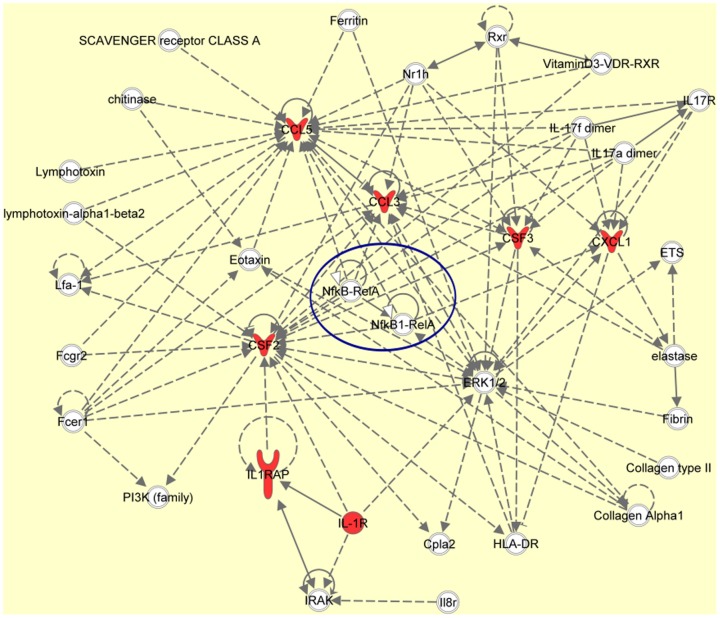
Molecular network showing cytokine and chemokine secretomes of human astrocytes. Entrez Gene IDs corresponding to 14 upregulated and 2 downregulated cytokines in IL-1β/TNF-α-activated human astrocytes were imported into the Ingenuity Pathways Analysis (IPA). The most significant molecular network relevant to the imported genes (red arrows) is shown. NF-kB is highlighted by blue circle. CCL5 (RANTES), CCL3 (MIP-1α), CXCL1 (GROα), CSF2 (GM-CSF), CSF3 (G-CSF), IL1R and IL1RAP are indicated by red.

When the list of Entrez Gene IDs corresponding to 14 up-regulated and 2 down-regulated cytokines in IL-1β/TNF-α-activated human astrocytes (Tables 4 and 5, Figs. 2 and 3) were imported into IPA, we identified the molecular network defined by “Cell-to-Cell Signaling and Interaction, Hematological System Development and Function, Immune Cell Trafficking” as the most significant functional network relevant to the set of imported genes (p = 1.00E-13) (Table 7, Fig. 4). The network defined by “Cellular Movement, Hematological System Development and Function, Immune Cell Trafficking” (p = 1.00E-6) was the second rank significant functional network (Table 7). IPA also indicated that nuclear factor NF-kB/RelA serves as an upstream regulator of the imported genes (p = 7.36E-24), validating the results of database search on NF-kB target genes described above. In IPA analysis, up-regulated molecules, such as MIP-1α (CCL3), RANTES (CCL5), GM-CSF, sICAM1, IL-1β, IL-6, IL-8 (CXCL8), and TNF-α, and a down-regulated molecule MCP-1 (CCL2), were categorized into NF-kB target genes located in the NF-kB signaling pathway (Fig. 4).

**Table 7:** Top 3 molecular networks of cytokine and chemokine secretome in human astrocytes.

Rank	Functional Networks	Focused Molecules	p-Value
1	Cell-To-Cell Signaling and Interaction, Hematological System Development and Function, Immune Cell Trafficking	**CCL3**, **CCL5**, **CSF2**, **CSF3**, **CXCL1**, chitinase, Collagen Alpha1, Collagen type II, Cpla2, elastase, Eotaxin, ERK1/2, ETS, Fcer1, Fcgr2, Ferritin, Fibrin, HLA-DR, IL-17f dimer, **IL-1R**, IL17a dimer, IL17R, IL1RAP, Il8r, IRAK, Lfa-1, Lymphotoxin, lymphotoxin-alpha1-beta2, NfkB-RelA, NfkB1-RelA, Nr1h, PI3K (family), Rxr, Scavenger receptor class A, VitaminD3-VDR-RXR	1.00E-13
2	Cellular Movement, Hematological System Development and Function, Immune Cell Trafficking	26 s Proteasome, Akt, AMPK, BCR (complex), calpain, **CCL2**, Cdk, Collagen type IV, Cyclin A, Cyclin E, cyclooxygenase, Fibrinogen, gelatinase, **GM-CSF**, Growth hormone, HDL, Ige, Ikb, JINK1/2, Laminin, LDL, **MIF**, N-cor, NADPH oxidase, Nos, PDGF (complex), PDGF BB, PRKAA, Ptk, Rb, Rock, **Serpine1**, Smad, Sphk, TGF-beta	1.00E-06
3	Infectious Disease, Cell-To-Cell Signaling and Interaction, Cellular Growth and Proliferation	Calcineurin protein(s), CD3, collagen, CYP, estrogen receptor, Hdac, hemoglobin, Histone h4, Hsp27, Hsp70, Hsp90, Icam, Iga, IgG1, Igm, **IL-6**, **IL-8**, Immunoglobulin, Interferon alpha, Ldh, Mek, Nfat (family), Notch, P38 MAPK, p70 S6k, Pro-inflammatory Cytokine, Rap1, Rsk, Serine Protease, Sod, SRC (family), STAT5a/b, **TNF**, TSH, U1 snRNP	1.00E-05

Functional networks were studied by importing Entrez Gene IDs of 14 up-regulated and 2 down-regulated cytokines in IL-1β/TNF-α-activated human astrocytes into the core analysis tool of IPA. They are listed with functional networks, focused molecules, and p-value of the Fisher's exact test. The first rank network is illustrated in Fig. 4.

## Discussion

Due to the limited availability of human brain tissues, only a small number of studies have previously reported the cytokine production profiles of normal human astrocytes [9]–[11]. In the present study, we characterized the more comprehensive profile of cytokine and chemokine named “secretome” derived from non-stimulated resting and activated human astrocytes by using a protein microarray. We found that both non-stimulated and IL-1β/TNFα-activated astrocytes produce distinct sets of cytokines and chemokines, nearly all of which represent direct targets of transcription factor NF-kB. One exception is MCP-1 (CCL2), a direct target of NF-kB [12], was down-regulated in activated astrocytes following exposure to IL-1β and TNF-α. In contrast, a previous study has indicated that the activated adult human astrocytes in culture produced increased amounts of MCP-1 [11]. The discrepancy between our results and previous findings is attributable to a difference in maturation of cultured cells, i.e. fetal versus adult astrocytes employed. These results suggest that the NF-kB signaling pathway differentially regulates gene expression of cytokines and chemokines in human astrocytes under physiological and inflammatory environments.

NF-kB acts as a central regulator of innate and adaptive immune response, stress response, cell proliferation, and apoptosis [13]. The NF-kB family proteins consist of five members, such as RelA (p65), RelB, c-Rel, NF-kB1 (p105), and NF-kB2 (p100). The latter two are processed proteolytically into p50 and p52, respectively. The NF-kB family proteins constitute either homodimers or heterodimers, except for RelB that exclusively forms heterodimers. The NF-kB dimers interact with consensus DNA sequences termed the kB site located on promoters to activate or repress transcription of target genes. Only p65 and c-Rel act as a potent transcriptional activator, whereas p50 and p52 homodimers generally repress transcription, leading to differential regulation of gene expression of NF-kB targets [14]. We found that MCP-1 (CCL2), a target of NF-kB, is down-regulated in NF-kB-activated human astrocytes. Importantly, NF-kB target genes often activate NF-kB itself, providing a positive regulatory loop that amplifies and perpetuates inflammatory responses [15]. IL-1 β and TNF-α are the prototypes of NF-kB activators for the canonical NF-kB signaling pathway. We found that non-stimulated human astrocytes do not constitutively produce IL-1β or TNF-α, while activated human astrocytes could produce both, as described previously [9], [11]. In contrast, a previous study has shown that IL-1β is undetectable at both mRNA and protein levels in non-stimulated or cytokine-stimulated cultured human astrocytes [10]. A different study from the same group showed that IL-8 (CXCL8) is undetectable in non-stimulated human fetal astrocytes in culture [16], being inconsistent with our results. In our study, non-stimulated astrocytes expressed a panel of NF-kB targets, such as G-CSF, GM-CSF, IL-6, GROα (CXCL1), IL-8 (CXCL8), MCP-1 (CCL2), MIF and serpin E1, suggesting that the NF-kB signaling pathway is constitutively active to a certain extent in normal human astrocytes in culture. Notably, GM-CSF serves as an anti-apoptotic and neurotrophic factor [17].

Chemokines constitutes a group of structurally related proteins that play a pivotal role in regulation of immune cell trafficking involved in inflammatory and immunoregulatory processes in the CNS [18]. In the present study, molecular network of cytokine and chemokine secretome of activated human astrocytes strongly supported the view that these soluble factors are critically involved in regulation of the cellular interaction and trafficking of immune cells. Various chemokine receptors, such as CCR2 with the ligand MCP-1 and CXCL2 with multiple ligands, such as Groα, Groβ and IL-8, are up-regulated in brain lesions of trauma, ischemia, and multiple sclerosis (MS) [19]. MIP-1 belongs to a family of C-C chemokines with a potent chemotactic activity for neutrophils and other leukocytes, composed of two members MIP-1α (CCL3) and MIP-1β (CCL4), both of which exhibit very similar but not identical proinflammatory properties through binding differentially to the receptors CCR1, CCR4 or CCR5. The upregulated expression of MIP-1α and MIP-1β was identified in acute MS lesions [20]. However, we identified MIP-1α (CCL3) but did not detect the expression of MIP-1 β (CCL4) in non-stimulated or activated human astrocytes in culture, suggesting that the gene regulatory mechanism is different between MIP-1α and MIP-1 β, although both are directly regulated by NF-kB.

RANTES is a member of C-C chemokines involved in the pathogenesis of MS and HIV-1 encephalitis by binding to the receptors CCR1, CCR3 or CCR5 [21], [22]. A previous study has shown that fetal human astrocytes upon activation by IL-1β secretes a large amount of RANTES (CCL5) protein [23], supporting our observation that RANTES is the most prominently up-regulated chemokine in activated human astrocytes in culture. Importantly, RANTES plays a neuroprotective role in ischemic brain injury [24]. The expression of IP-10 (CXCL10), secreted by monocytes, endothelial cells, and fibroblasts in response to IFN-γ, shows a chemotactic activity for T cells, NK cells, dendritic cells and monocytes/macrophages through binding to the receptor CXCR3. We found that IP-10 production is greatly enhanced in activated human astrocytes in culture. Notably, the expression of IP-10 and CXCR3 is up-regulated in the brains of AD, where CXCR3 is expressed constitutively on neurons, while IP-10 expression is enhanced in a subset of reactive astrocytes surrounding senile plaques [25].

In conclusion, the comprehensive cytokine and chemokine secretome of activated human astrocytes, closely linked to NF-kB activation, suggested that astrocyte-derived cytokines and chemokines play a central role in proinflammatory (neurotoxic) and immunoregulatory (neuroprotective) responses in the CNS.
